# Transactions of the American Medical Association for 1875

**Published:** 1876-01

**Authors:** 


### BIBLIOGRAPHICAL.

*Transactions of the American Medical Association, Volume* 26, 1875.—The contents consist of the proceedings of the 26th annual meeting, held at Louisville, Kentucky, in May, 1875. During this session a large number of interesting papers were read, which appear in full in the present volume, rendering it an exceedingly instructive and interesting publication. These reports of the various committees, although formally accepted and published, are independent, the Association holding itself wholly irresponsible for the opinions, theories, or criticisms therein contained, except when otherwise decided by special resolution. The present volume contains five-hundred and twenty-seven pages.

